# Simulated Flock-Level Shedding Characteristics of Turkeys in Ten Thousand Bird Houses Infected with H7 Low Pathogenicity Avian Influenza Virus Strains

**DOI:** 10.3390/v13122509

**Published:** 2021-12-14

**Authors:** Peter J. Bonney, Sasidhar Malladi, Amos Ssematimba, Kaitlyn M. St. Charles, Emily Walz, Marie R. Culhane, David A. Halvorson, Carol J. Cardona

**Affiliations:** 1 Secure Food Systems Team, Department of Veterinary and Biomedical Sciences, College of Veterinary Medicine, University of Minnesota, Saint Paul, MN 55108, USA; malla042@umn.edu (S.M.); assemati@umn.edu (A.S.); stcha003@umn.edu (K.M.S.C.); walzx148@umn.edu (E.W.); grame003@umn.edu (M.R.C.); halvo002@umn.edu (D.A.H.); ccardona@umn.edu (C.J.C.); 2 Department of Mathematics, Faculty of Science, Gulu University, Gulu P.O. Box 166, Uganda

**Keywords:** avian influenza, epidemiology, mathematical modeling, outbreak management, poultry

## Abstract

Understanding the amount of virus shed at the flock level by birds infected with low pathogenicity avian influenza virus (LPAIV) over time can help inform the type and timing of activities performed in response to a confirmed LPAIV-positive premises. To this end, we developed a mathematical model which allows us to estimate viral shedding by 10,000 turkey toms raised in commercial turkey production in the United States, and infected by H7 LPAIV strains. We simulated the amount of virus shed orally and from the cloaca over time, as well as the amount of virus in manure. In addition, we simulated the threshold cycle value (Ct) of pooled oropharyngeal swabs from birds in the infected flock tested by real-time reverse transcription polymerase chain reaction. The simulation model predicted that little to no shedding would occur once the highest threshold of seroconversion was reached. Substantial amounts of virus in manure (median $1.5\times 10^{8}$ and $5.8\times 10^{9}$; 50% egg infectious dose) were predicted at the peak. Lastly, the model results suggested that higher Ct values, indicating less viral shedding, are more likely to be observed later in the infection process as the flock approaches recovery.

## 1. Introduction

Low pathogenicity avian influenza (LPAI) can cause reduced egg production and weight gain in infected birds, cause increased flock mortality, and impact the trade of poultry and poultry products [1]. H5 and H7 LPAI virus (LPAIV) strains are of special concern, as they have been known to mutate into highly pathogenic avian influenza (HPAI), a devastating virus that can cause close to 100% mortality in an infected flock. Historically, LPAI outbreak responses in the United States (U.S.) have used one of three strategies: depopulation (on-site destruction of the birds on infected premises), controlled marketing (maintaining birds on premises with heightened biosecurity until birds recover and are sent to processing), and, though less common in the U.S., vaccination [1,2].

LPAIV has been documented to be spread by activities and movements of people, especially due to direct contact with infected manure [1]. As such, heightened levels of activity on a confirmed LPAI-infected premises can increase risk of virus transmission. Halvorson, (2008) stresses the need to “do no harm” following detection of LPAI, and provides case reports where depopulation was implicated in virus spread [1,3,4]. In order to “do no harm,” the outbreak response must be carefully considered prior to any action taken. The decision of whether to depopulate, do controlled marketing, or vaccinate depends on many factors, including flock production considerations, local and regional outbreak status, and biosecurity. This is a decision that must be carefully discussed between state and industry partners, and is most easily reached when each has a well-thought-out LPAI outbreak plan.

An important tool for outbreak response is diagnostic testing by real-time reverse transcription polymerase chain reaction (rRT-PCR), which can be used for detection and monitoring of avian influenza virus in infected flocks [5]. Pooled samples of oropharyngeal (OP) swabs from 11 birds tested by rRT-PCR are a common suggestion for surveillance. Spackman et al. (2013) explain that 11 OP swabs from a flock of 10,000 or more birds has been determined to be sufficient to achieve the National Poultry Improvement Plan target of detecting 25% infection prevalence with 95% confidence [6]. The threshold cycle (Ct) value derived from rRT-PCR testing can indicate the amount of virus in the sample. Higher Ct values are associated with lower amounts of virus, and for most laboratories, Ct values < 40 are considered positive [7]. Birds infected with LPAIV typically shed lower amounts of virus as they approach recovery, and have weak-positive or negative LPAIV rRT-PCR results with Ct values that are often between 35 and 40 [8,9].

Though it is understood that the individual birds in an infected poultry house shed large amounts of virus, to our knowledge, no attempt has been made to quantify this amount for LPAIV-infected poultry flocks. Only rarely has the amount of virus shed by an infected flock been modeled for avian diseases in general [10]. Therefore, we developed mathematical models to analyze flock shedding dynamics. The model was developed based on largescale commercial turkey houses in the U.S., and H7 LPAIV strains. We simulated the amount of oral and cloacal viral shedding with a stochastic disease transmission model for a poultry house containing 10,000 turkey toms.

Viral shedding was simulated at the individual bird level, and allowed to vary over time, diverging from the assumption in classical epidemiological models of constant shedding during the infectious period [11]. The viral shedding model was extended to (1) approximate the amount of virus in manure over time, and (2) estimate the likelihood of observing higher (>30 and ≤40) and lower (≤30) threshold cycle (Ct) values following testing of 11 OP swabs sampled from turkeys in the house by rRT-PCR. This was done to investigate whether having LPAIV rRT-PCR results with Ct values approaching 40 could be used as a flock-level indicator that a previously positive flock is nearing recovery. The models were developed to provide insight into the transmission risk that infected flocks pose following LPAIV exposure, and to inform appropriate outbreak response strategies in order to minimize the risk of LPAI spread to other flocks.

## 2. Materials and Methods

### 2.1. Simulation of Virus Shedding over Time in a Turkey Flock

We simulated the amount of LPAIV shed in a house of turkey toms via oral and cloacal routes over time using an individual-based stochastic disease transmission model. The transmission model consisted of (1) a compartmental component that tracked the number of birds in the following disease states: susceptible, latently infected, infectious, seroconverted, dead, or recovered per simulation time step (set to 0.01 days); (2) a component that simulated the amount of virus shed from oral and cloacal routes by infected birds per simulation time step; and (3) a component that simulated the amount of infectious virus in manure.

### 2.2. Simulation of Disease State Transitions

Specific methodology and parameterization of the compartmental component was implemented as described in [12]. The outbreak was assumed to begin with a single latently infected bird. The transition of turkeys from the susceptible to the latently infected state was simulated from a binomial distribution where the probability of infection depended on the adequate contact rate (transmission rate parameter), and proportion of infectious and alive (susceptible, latently infected, infectious, and recovered) birds. Random numbers were generated from distributions modeling the length of the latent, and infectious periods for each infected bird that determined the transition times out of the latent and infectious states. Birds transition from the infectious state into either the recovered or dead state according to a binomial distribution. The number of infected turkeys that seroconvert, i.e., develop detectable antibodies, was also simulated from a binomial distribution. The transition of infected birds into the seroconverted state was determined by a random number generated from a distribution modeling the time between infection and seroconversion. A summary of the algorithm is given by Algorithm 1.
**Algorithm 1:** Algorithm to simulate the number of birds in different disease states1Initialize a latently infected bird2Create arrays for the number of birds per time step in the disease states latently infected, infectious, seroconverted, recovered, and dead3for all simulation time steps do4
Simulate the number of birds infected during the previous time step5for all newly infected birds do6
Simulate the number of time steps the bird is in the latent period7Simulate the number of time steps the bird is in the infectious period8Simulate whether the bird dies or recovers9Simulate whether or not the bird seroconverts10if the bird seroconverts then11
Simulate the number of time steps until the bird seroconverts12end13Update disease state arrays14end15end

The length of the latent and infectious periods, and time to seroconversion were all assumed to be gamma distributed. The shape and scale parameters of the gamma distributions were estimated using a maximum likelihood estimation approach from data obtained from experimental inoculations of turkeys in controlled settings [8,9,13,14,15,16,17,18,19,20]. The experiments used to estimate the latent and infectious period distributions involved H5 and H7 LPAIV strains [8,9,13,14,15,16]. The experiments used to estimate the seroconversion distribution involved H4, H6, H7, and H9 LPAIV strains, due to insufficient H5 and H7 antibody data [17,18,19,20]. The proportion of turkeys infected with LPAIV that die was set to 1% based on mortality observed during the 2018 LPAI H5N2 outbreak in Minnesota [21]. The proportion of turkeys that seroconvert was set to 99% based on the results of Spackman et al. (2010) [8]. The adequate contact rate for a simulation iteration was drawn from a uniform distribution with minimum 0.5, and maximum 4.0, based on the results of Bonney et al. (2021) [12]. A summary of the transmission model parameters is given in Table 1.

### 2.3. Simulation of Viral Shedding

Viral shedding from oral and cloacal routes was simulated using experimental data from two inoculation studies in order to capture potential differences in shedding between virus strains [8,9]. A different simulation approach was used for each study, depending on the data characteristics.

The first method involved selecting data directly from a turkey inoculation experiment performed by Iqbal et al. (2012) [9]. The data used from this experiment consisted of thirty turkeys that had been inoculated or placed with birds that had been inoculated with a H7N1 LPAIV strain collected in 1999 during an outbreak in Italy. Ten turkeys were inoculated with a dose of $10^{7.4}$ 50% egg infectious dose per milliliter ($EID_{50}/ml$), whereas ten naïve turkeys were placed with ten turkeys inoculated with a dose of $10^{3.8}\;EID_{50}/ml$. The $EID_{50}/ml$ unit of measurement represents the amount of virus that will infect 50% of inoculated eggs. Buccal and cloacal swabs were collected daily from the turkeys, and tested by quantitative RT-PCR (qRT-PCR) to obtain the Ct value. The Ct was converted to $log_{10}\;EID_{50}/ml$ using a standard curve [9].

The Iqbal et al. (2012) data were implemented into the transmission model as follows [9]. First, the data were prepared for use in the model. The amount of buccal and cloacal shedding per simulation time step was estimated by linear interpolation between observed data points. Contact turkeys were assumed to be infected one day prior to the first positive test. In the transmission model, for each infected turkey, the shedding pattern of an individual bird was selected at random from the data. Once the turkey transitioned into the infectious period, the selected oral and cloacal shedding pattern per simulation time period was added to the existing amounts to estimate a total for all the birds in the house.

In the second method, viral shedding was simulated from regression models fit to turkey inoculation data from Spackman et al. (2010) [8]. Spackman et al. (2010) inoculated turkeys with twelve North American H7 LPAIVs, where 13 to 15 turkeys were inoculated per strain [8]. Oropharyngeal (OP) and cloacal swabs were taken 2, 4, 7, 10, and 14 days post inoculation, and tested by qrRT-PCR. The Ct values were converted to $log_{10}\;EID_{50}/ml$ using standard curves [8].

The experimental data were zero inflated because many birds did not shed virus every day post inoculation. Therefore, two-part models were fit to the OP and cloacal swab data to accommodate the intermittent shedding observed in the experiment. In a two-part model, the observed data is treated as a mixture of the zero and nonzero components [22]. In the context of the Spackman et al. (2010) data, the probability that a bird does not shed at a given time post inoculation, and the amount of virus that a bird sheds, given the amount is not zero, were estimated by mixed-effects regression models [8,23].

Several regression models were evaluated, varying by the inclusion of a quadratic term for the number of days post exposure, and random intercept and slope terms for each virus strain [24,25,26]. Models were selected according to the Akaike Information Criterion (AIC) where differences of less than 2 were not considered significant, per the suggestion of Burnham and Anderson (2002) [27,28]. Though the amount of virus shed by individual birds is likely correlated between time points, this information was not available in the data.

The two-part model selected from the cloacal swab data had the following form. The probability that a bird *i* inoculated with virus strain *j* did not shed at time $t_{ij}$ was given by:

(1) $$logit\left\{P\left(y_{ij}=0\right)\right\}=\beta_{0}+\beta_{1}t_{ij}+\beta_{2}t_{ij}^{2}+b_{0j}+b_{1j}t_{ij},$$

where $y_{ij}$ is the $log_{10}\;EID_{50}/ml$ observed for bird *i* inoculated with virus *j*; $\beta_{0}$, $\beta_{1}$, and $\beta_{2}$ are the fixed effects; $b_{0j}$ is the random intercept, and $b_{1j}$ is the random slope for virus *j* with $\left\{b_{0j},b_{1j}\right\}\;\sim \;N\left(0,D\right)$, where D is the variance-covariance matrix. Given bird *i* inoculated with virus strain *j* sheds virus at time $t_{ij}$, the amount in $log_{10}\;EID_{50}/ml$, $y_{ij}$ was given by:

(2) $$y_{ij}|y_{ij}>0=\beta_{0}^{*}+\beta_{1}^{*}t_{ij}+\beta_{2}^{*}t_{ij}^{2}+b_{0j}^{*}+b_{1j}^{*}t_{ij}+\varepsilon_{ij},$$

where $\beta_{0}^{*}$, $\beta_{1}^{*}$, and $\beta_{2}^{*}$ are the fixed effects; $b_{0j}^{*}$ is the random intercept, and $b_{1j}^{*}$ is the random slope for virus *j* with $\left\{b_{0j}^{*},b_{1j}^{*}\right\}\;\sim \;N\left(0,\;D^{*}\right)$, where $D^{*}$ is the variance-covariance matrix; and $\varepsilon_{ij}$ is the random error with $\varepsilon_{ij}\;\sim \;N\left(0,\;\sigma^{2}\right)$. The two-part model selected from the OP swab data was similar, except for the exclusion of the random slope term in Equation (1). The random slope term was excluded since its inclusion resulted in a higher AIC value as compared to the model with only a random intercept. Parameters were estimated using the lme4 and nlme packages in R [29,30,31]. Further details on the two-part regression models are given in Appendix A.

The regression equations were implemented into the transmission model by first randomly selecting a virus strain for the iteration. Once a turkey transitioned into the infectious state, the amount of virus shed from OP and cloacal routes was simulated from the appropriate regression equations for each simulation time step until 21 days post inoculation, by which point, birds would be expected to have stopped shedding [32]. The algorithm used to simulate oral and cloacal shedding over time by turkeys infected with LPAIV is summarized by Algorithm 2.
**Algorithm 2:** Algorithm to simulate the amount of viral shedding from oral and cloacal routes1Create arrays for the amount of virus shed by oral and cloacal routes in each simulation time step2for all simulation time steps do3 for all birds infected in previous simulation time step do4  if Iqbal et al. (2012) scenario then5   Randomly select a bird from the experimental data6   for all data time steps do7    if simulated bird has transitioned out of latent period and is alive then8     Update viral shedding arrays at simulation time step + data time step using the selected experimental data9    end10   end11  end12  if Spackman et al. (2010) scenario then13   for all time steps between 0 and 21 days do14    if bird has transitioned out of latent period and is alive then15     Simulate using two-part model whether or not bird sheds orally16     if bird sheds orally then17      Simulate amount of oral shedding using two-part model18      Update oral shedding array at simulation time step + day time step19     end20     Simulate using two-part model whether or not bird shed from the cloaca21     if bird sheds from the cloaca then22      Simulate the amount of cloacal shedding using the two-part model23      Update cloacal shedding array at simulation time step + day time step24     end25    end26   end27  end28 end29end

### 2.4. Simulation of the Amount of Virus in Manure

The $log_{10}\;EID_{50}/ml$ of virus shed from cloacal routes simulated for individual birds based on the Iqbal et al. (2012) and Spackman et al. (2010) data was translated into the virus titer in manure by multiplying the exponentiated simulated $log_{10}\;EID_{50}/ml$ by the average volume of feces produced in a simulation time period [8,9]. The milliliters of feces excreted in a simulation time period was approximated based on mean feed consumption by weight of adult turkey toms between 16 and 20 weeks of age [33]. The weight of feed was converted to volume of waste produced using a 1:1 conversion ratio for the mass of waste produced per mass of feed consumed [33], and the feces density derived from [34].

In addition to the amount of virus present in the manure produced during a given time period, the virus titer in the cumulative amount of feces produced over time was estimated as well. This was done for time *t* by adding the virus titer of feces newly produced in the simulation time period *t* to the cumulative amount of virus estimated for time *t-1* reduced by a scaled decay rate. The decay rate was derived from experimental data from Wood et al. (2010) and Lu et al. (2003), and estimated as the decrease in $log_{10}\;EID_{50}/ml$ of virus in feces per day [35,36]. The rates taken from Lu et al. (2003) involved the decay of a LPAIV H7N2 strain in feces from a specific pathogen-free chicken stored at 15–20 °C, feces from an industry-raised chicken stored at 28–30 and 15–20 °C, and feces sampled directly from a commercial flock stored at 28–30 °C [36]. The selected Wood et al. (2010) scenarios involved the decay of an HPAIV H5N1 strain in feces stored at 23 °C in conditions of low and high relative humidity [35]. The algorithm used to simulate the amount of LPAIV in manure is summarized by Algorithm 3.
**Algorithm 3:** Algorithm to simulate the amount of virus in manure1Create an array for the amount of virus in feces produced in a simulation time step2Create an array for the cumulative amount of virus in feces in a simulation time step3for all simulation time steps do4 for all birds infected in previous simulation time step do5  Simulate cloacal shedding for a bird according to Algorithm 26  Estimate the amount of virus in feces produced by the bird in the time step (amount of virus per ml × ml feces produced)7  Update the array with the amount of virus produced in a simulation time step8 end9 Estimate the cumulative amount of virus in feces in the simulation time step (cumulative amount from previous time step × decay rate + amount of virus in feces produced in current time step)10 Update the array with the cumulative amount of virus in feces in a simulation time step11end

### 2.5. Simulation of Ct Values from OP Swabs Tested by rRT-PCR

The expected Ct value from a pooled sample of OP swabs tested by rRT-PCR was simulated from the amount of viral shedding from oral routes in individual birds estimated from the transmission model as an exploratory analysis. In the disease transmission model, the simulated amount of oral shedding of an individual bird was converted into a Ct value using a standard curve from Spackman et al. (2010) [8]. The Ct values were then sorted into groupings of values to estimate the number of birds over time expected to have certain Ct values. The Ct values were stored in this way to ease computational demands. The Ct values were grouped as follows: 10–14, 14–18, 18–22, …, 34–38, or 38–40. The Ct value of birds that died due to LPAI was set equal to the value simulated in the time step immediately prior to death.

The likelihood of observing a Ct value ≤ 30 (i.e., a strong positive), and the likelihood of observing a Ct value > 30 but ≤40 (i.e., a weak positive) over time following the testing of a pooled sample of 11 OP swabs by rRT-PCR were estimated using forward simulation. The application of the testing protocol was simulated at each 0.25 day from the time of virus exposure until 80 days post exposure for 6000 transmission model iterations. The likelihoods were determined by the proportion of iterations with simulated Ct values falling within the desired ranges. The testing protocol consisted of a single pooled sample of 11 OP swabs. Dead birds were prioritized for sampling. If fewer than 11 birds had died, samples were taken from live birds until the total of 11 swabs was reached.

To simulate the application of the testing protocol, first daily mortality was estimated by summing the normal mortality, simulated from industry data using the approach described in Weaver et al. (2016), and mortality due to LPAI, taken from the transmission model output [37]. Three sources of positive swabs were considered: birds that died directly due to LPAI, birds that died while infected (but not due to infection), and infected live birds. The number of birds that died while infected, but not due to infection, was simulated from the normal mortality where the probability a dead bird was infected was set equal to the infection prevalence in the flock. The number of swabs from infected birds in the daily mortality was simulated from a hypergeometric distribution. The number of swabs from infected live birds was simulated from a binomial distribution with the probability of success equal to the infection prevalence at the time of sampling taken from transmission model output. If a swab from at least one positive bird was included in the pooled sample, the test outcome was simulated as a Bernoulli trial with probability of success equal to the test sensitivity, here set to 0.90 [37,38].

A Ct value was simulated for each positive simulated rRT-PCR result. The amount of virus in the sample contributed by each swab from a positive bird was estimated by first simulating the number of swabs belonging to each Ct value range (10–14, 14–18, etc.). This was simulated from a multinomial distribution where the probabilities were determined by the number of birds in each category (as simulated from the transmission model) divided by the total across all categories at the time of sampling. For each swab in a certain Ct value range, the exact value was simulated from a uniform distribution. The Ct values were then converted back into $log_{10}\;EID_{50}/ml$ using the standard curve. The amount of virus in the pooled sample was assumed to be additive, that is, the $log_{10}\;EID_{50}/ml$ amounts were exponentiated and summed. In the last step, the sum was converted into a Ct value with the standard curve from Spackman et al. (2010) [8]. The algorithm is summarized in Algorithm 4.
**Algorithm 4:** Algorithm to simulate Ct value of 11 pooled OP samples tested by rRT-PCR Simulate Ct values of OP swab samples from individual birds:1Create arrays to store the number of birds in each simulation time point that if swabbed and tested would have a Ct value within a certain range (e.g., Ct value between 10 and 14, 14 and 18, etc.)2for all simulation time steps do3 for birds infected in previous simulation time step do4  Simulate oral shedding for a bird according to Algorithm 25  Convert the oral shedding amounts to Ct values using a standard curve6  Update the Ct value range arrays7 end8end Simulate selecting birds for OP swabs and testing pooled sample by rRT-PCR:9Simulate normal daily mortality10Simulate mortality due to LPAIV and infection prevalence over time according to Algorithm 111for all simulation time steps do12 Simulate the number of birds from the normal daily mortality that died while LPAI-positive based on the infection prevalence in the flock13 Simulate the number of swabs taken from LPAI-positive and LPAI-negative dead birds 14 if the total number of dead birds is less than 11 then15  Simulate the number of swabs taken from LPAI-positive and LPAI-negative living birds based on the infection prevalence in the flock16 end17 if there was at least one swab from a LPAI-positive bird then18  Simulate the rRT-PCR test outcome19  if the rRT-PCR test result is positive then20   for all swabs from LPAI-positive birds do21    Simulate the Ct range of the swab using the Ct value range arrays22    Simulate the specific Ct value from between the selected range23    Convert the Ct value to $EID_{50/ml}$ using a standard curve24   end25   Sum the simulated virus amounts of the swabs from LPAI-positive birds26   Convert the virus amount to a Ct value using a standard curve27  end28 end29end

The models were programmed and run using R statistical software, and C programming language [39,40]. The simulations were run for 6000 iterations for a flock size of 10,000 turkey toms.

## 3. Results

Epidemiological outcomes (seropositivity) estimated from the disease transmission model are given in Table 2. Median estimates and 95% prediction intervals are given for the number of days post virus exposure, infection prevalence, and the proportion of the cumulative oral and cloacal shedding from both datasets reached when the given seropositive thresholds are first surpassed. The highest seropositive threshold was set to 98% due to variance in the number of birds that seroconvert following infection, which was given a 99% probability of occurring [8]. As expected, the number of seropositive birds increased over time, with the 98% seropositive threshold reached 3–6 weeks post virus exposure. Peak infection prevalence was predicted to occur around 30% seroconversion, before dropping down to a median of 23% when 98% seropositive birds was first reached. The shedding by infectious birds when the 98% seropositive threshold was first reached was at post-peak amounts: the median across all simulation iterations of the average amount of shedding by infectious birds at the time the 98% seropositive threshold was first reached was 2.46 $log_{10}\;EID_{50}/ml$ for oral shedding, and 3.06 $log_{10}\;EID_{50}/ml$ for cloacal shedding in the Spackman et al. (2010) scenario; whereas in the Iqbal et al. (2012) scenario, the amount was 1.05 $log_{10}\;EID_{50}/ml$ for oral shedding, and 0.22 $log_{10}\;EID_{50}/ml$ for cloacal shedding [8,9]. The amount of virus shed by infectious birds subsequent to this time accounted for very little of the total: Approximately all shedding from oral and cloacal routes was predicted to have occurred by the time 98% seroconversion was observed for both datasets. Peak cloacal shedding was predicted to occur after peak oral shedding, an effect more pronounced in the Spackman et al. (2010) as compared to the Iqbal et al. (2012) scenario [8,9].

**Figure 1:**
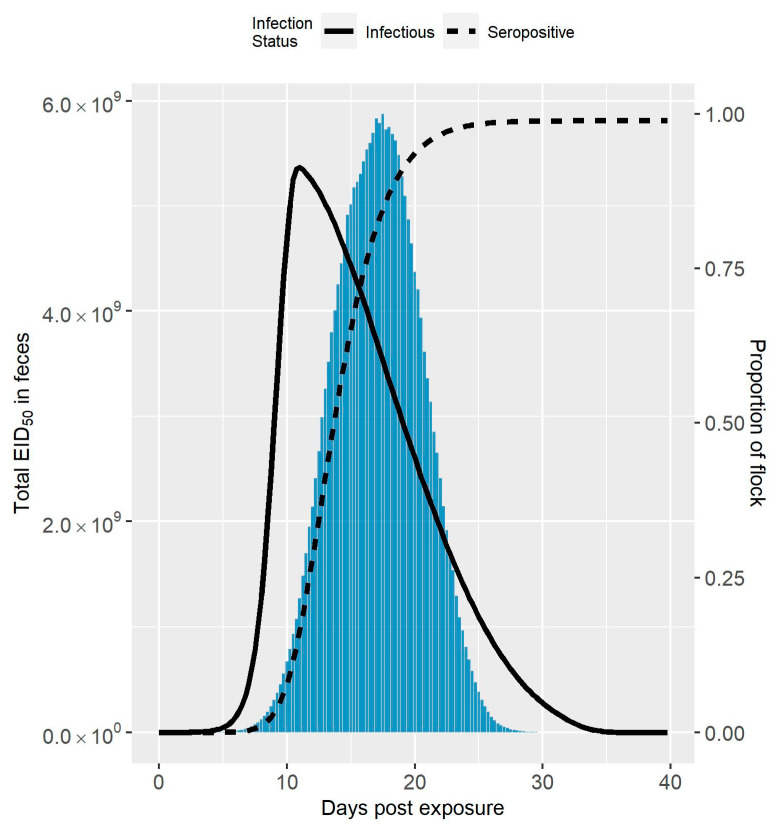
Amount of LPAIV in Feces Simulated from the Spackman et al. (2010) Data. Median total amount of LPAIV in feces over time simulated for a 10,000 bird turkey tom flock from the Spackman et al. (2010) data compared to the seroprevalence and infection prevalence in the flock [8]. The amount of virus is represented by the bars, whereas the seroprevalence and infection prevalence are represented by the dashed and solid lines, respectively.

**Figure 2:**
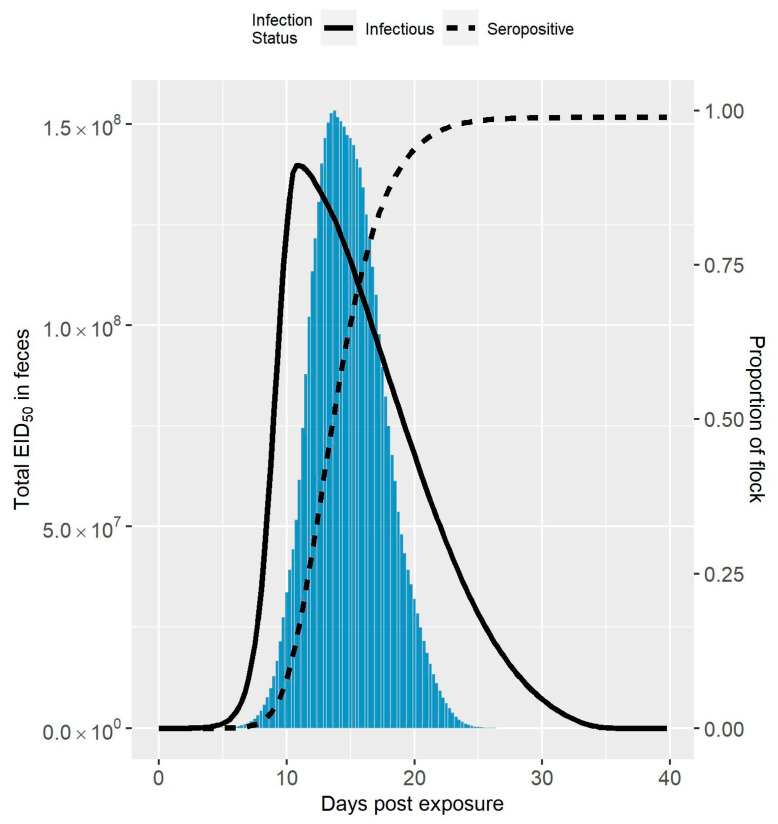
Amount of LPAIV in Feces Simulated from the Iqbal et al. (2012) Data. Median total amount of LPAIV in feces over time simulated for a 10,000 bird turkey tom flock from the Iqbal et al. (2012) data compared to the seroprevalence and infection prevalence in the flock [9]. The amount of virus is represented by the bars, whereas the seroprevalence and infection prevalence are represented by the dashed and solid lines, respectively.

The median amount of virus in feces over time following virus exposure compared to the median seroprevalence and infection prevalence is shown in Figure 1 for Spackman et al. (2010), and Figure 2 for Iqbal et al. (2012) [8,9]. The maximum amount of virus was an order of magnitude larger in the Spackman et al. (2010) as compared to the Iqbal et al. (2012) scenario ($5.8\times 10^{9}\;EID_{50}$ compared to $1.5\times 10^{8}\;EID_{50}$). In both cases, the peak amount of virus was predicted to occur after the peak infection prevalence. This time lag was more pronounced in the Spackman et al. (2010) data, leading to substantial shedding occurring when large numbers of birds were predicted to be seroconverted. Virus was still predicted to be present at the lower levels of infection prevalence early and late in the infection process, but in amounts that were dwarfed by the amounts observed at the peak, and, therefore, are not visible in Figure 1 or Figure 2.

**Figure 3:**
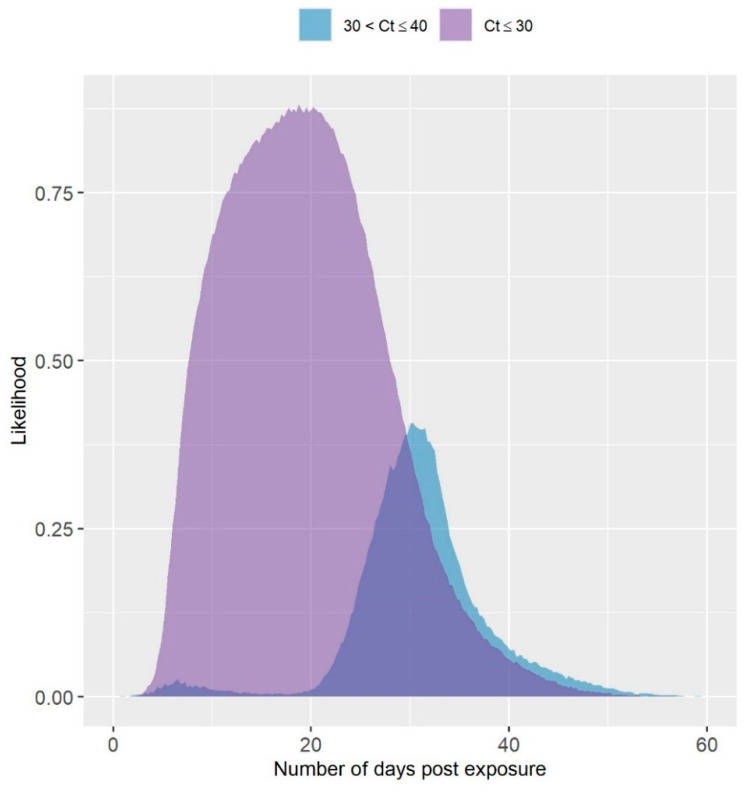
Simulated Likelihood of Observing a High or Low Ct Value from a Pooled Sample of 11 OP Swabs. The likelihood of observing a Ct value less than or equal to 30, or between 30 and 40 over time in a 10,000 bird turkey tom house based on a testing protocol of one pooled sample of 11 OP swabs collected from dead birds first, and then live birds as needed.

The likelihood of observing a Ct value of ≤30 and >30 but ≤40 following exposure of a turkey tom house to LPAIV is given in Figure 3. The likelihood was estimated for a testing protocol of 11 OP swabs pooled and tested by rRT-PCR. The likelihood of obtaining a Ct value > 30 was estimated to be highest several weeks after exposure. The peak likelihood of obtaining a Ct value < 30 was comparably earlier by about 10 days. Ct values below 30 were estimated to have a much higher likelihood, in general. Though the likelihood of observing a Ct value above 30 was estimated to occur primarily later in the infection, there was a slightly elevated likelihood predicted approximately seven days post exposure.

## 4. Discussion

In this analysis, the amount of virus shed from oral and cloacal routes over time by ten thousand turkey toms in a house exposed to LPAIV was simulated from a stochastic disease transmission model, parameterized based on largescale production in the U.S., to provide insight into the risk of disease spread posed by infected flocks over time. The results suggest that oral shedding more closely relates to infection prevalence than cloacal shedding, which occurs later in the disease course. This is consistent with the experimental data used from Spackman et al. (2010), and Iqbal et al. (2012), in which peak cloacal shedding occurred after peak oral shedding in individual birds [8,9]. See [41] for more discussion.

The disposal of infected birds by controlled marketing or destruction must be carefully considered. Transporting actively shedding birds off-farm poses a substantial risk to poultry premises located along the route, due to contaminated feathers and feces dropped from the truck [42]. Similarly, destruction of actively shedding birds on-site can create opportunities to spread infectious material that poses a risk to nearby premises [4]. Depopulation activities can bring large amounts of people and equipment onto the premises, which may also pose transmission risk [1,3].

In this analysis, nearly all shedding was predicted to have occurred by the time the highest seropositive threshold was reached (see Table 2). This suggests that a diagnostic test result of 100% seropositive serum samples could provide evidence that the flock is at or close to recovery. The confidence that a flock is at or near recovery could be further improved with rRT-PCR testing. In particular, the results in Figure 3 predict that higher Ct values are more likely at the later stages of infection in the flock.

Though diagnostic testing can improve confidence that the amount of viral shedding in an infected flock is low, it should be a part of a more comprehensive strategy that includes other risk mitigation measures, such as monitoring the flock for clinical signs, and heightened biosecurity. The analysis provided here could inform a broader risk-based analysis related to decision-making regarding outbreak response, specifically in relation to the disposal of the exposed flock(s).

Halvorson (2008) identified that live avian influenza virus generally cannot be detected two weeks after the peak of clinical signs in past applications of controlled marketing [1]. Assuming the clinical signs peak is highly correlated with the peak of infection prevalence, this observation is fairly consistent with the predicted amount of virus in feces shown in Figure 1 and Figure 2. As can also be seen in Figure 1 and Figure 2, the peak amount of virus is considerable. Knowing the infection status of the flock in relation to the peak amount of shedding would be advantageous for deciding on the outbreak response, for example when and how to dispose of the birds. However, detection can occur at any time, and it can be difficult to know with certainty where the flock is in the infection process initially following detection.

The spread of LPAIV within a turkey house is an incredibly complex process subject to numerous variables, including the virus strain, environmental conditions, housing structure, and production practices. Due to the number of variables involved in LPAIV spread, the results presented here likely do not represent the diversity of outcomes that may be observed in the field. The analysis conducted here is meant to be an example or approximation of the LPAIV spread that may be observed in largescale turkey production in the U.S. based on available experimental and outbreak data.

The data used in the analysis involved unvaccinated turkeys not infected previously with LPAIV. Vaccination can reduce the amount and duration of viral shedding, as well as increase the minimum infectious dose necessary for a bird to become infected [43]. Therefore, the flock-level virus shedding characteristics of vaccinated turkeys or turkeys exposed to LPAIV in the past would likely be quite different from the results presented here.

To have sufficient data, a variety of sources involving different LPAIV strains were used. Consequently, this analysis does not capture the behavior of any one virus strain. As the amount of shedding was simulated based on experimental data involving H7 strains, the results may be regarded as for a generic H7 virus.

As a result of the variety and limitations of the sources used to parameterize the simulation model, the biological processes may not be captured perfectly. For example, though nearly all shedding was predicted to have occurred when the highest threshold of seroconversion was first achieved, there were still a considerable number of infectious birds at this time, an estimated median of 23%. In particular, substantial cloacal shedding was predicted at high levels of seroconversion in the Spackman et al. (2010) scenario [8]. In Table 1, at 80% seroconversion, a median of 58% of the cloacal shedding was estimated to have occurred, meaning 42% remained. Virus was detected post seroconversion in Preskenis et al. (2010), a study used to estimate the infectious period length and time to seroconversion distributions [20]. However, Preskenis et al. (2010), and the other studies used to estimate parameters for the transmission model, tested for or quantified the amount of virus using rRT-PCR [20]. Therefore, the model parameters are derived from data on the presence of virus, as opposed to the presence and viability of the virus. In addition, as discussed earlier, simulation inputs for the distributions for the length of the infectious and latent periods, the distribution for the time to seroconversion, and the amount of shedding over time were estimated independently, from an amalgamation of sources. In reality, these processes are likely correlated. Sufficient data was not available for joint estimation, but the associations between the responses to infection may not be captured as a result.

There are also uncertainties related to the estimation of the amount of virus in manure. A constant decay rate was used for the virus shed in feces in all previous time periods. However, the decay rate would likely be different between the newly excreted feces on top, and the buried feces excreted several days prior. For example, Beard et al. (1984) found avian influenza virus lasted one day longer in manure placed in vials with a cap (comparable to buried feces) as compared to vials without a cap (comparable to newly excreted feces) at 25 °C [44].

The amount of virus in feces over time is likely highly variable between flocks due to the dependence on multiple environmental factors, such as temperature and humidity [35,36]. It should be noted that the results given in Figure 1 and Figure 2 show the median amounts of virus in feces estimated from the simulation runs. Therefore, Figure 1 and Figure 2 may underrepresent the transmission risk posed by virus in feces for some cases. In the experience of Kradel (2003), during the 1983-84 LPAI/HPAI H5N2 outbreak in Pennsylvania, virus isolation was generally not successful 2 weeks after depopulation [3]. However, he notes that virus was recovered on five farms one month or more after depopulation [3].

Considerable uncertainty surrounds the effect of pooling multiple swabs from positive birds on the Ct value. Ladman et al. (2012), Arnold et al. (2013), and Spackman et al. (2013) explored the effect of pooling a single swab from chickens or turkeys inoculated with LPAI with swabs from uninfected birds on the resulting Ct value [6,45,46]. The results of these studies suggest pooling may affect the Ct value, but the nature of this effect remains unclear. Furthermore, no studies were identified at the time of writing that explored the effect of pooling swabs from more than one infected bird on the Ct value. The assumption made in the current analysis was that the amount of virus in the sample is additive per swab from an infected bird, with no diluting effect from swabs from uninfected birds. Although the uncertainty surrounding the effect of pooling on the Ct value exists, the likelihood of observing a pooled sample with Ct ≤ 30, or >30 and ≤40 resembled the makeup of the disease mortality. The estimated proportion of the disease mortality containing birds with Ct values greater than 30 increased with the number of days post exposure, which suggests that the likelihood of observing Ct values greater than 30 would follow a similar pattern under a testing program consisting of individual OP swab samples tested by rRT-PCR.

Though higher Ct values could be more likely later in the infection process, they are not confined to this time period. For example, a Ct value of 37.7 was observed over 30 days prior to the last positive test in a barn infected during the 2018 LPAI H5N2 outbreak in Minnesota [21]. However, Ct values in the thirties were more often observed closer to when house testing started to yield rRT-PCR negative results on these premises [21]. In Figure 3, a small bump in the likelihood of observing a Ct value between 30 and 40 was estimated less than 10 days following exposure. This could be due to the combination of low prevalence in the flock, and few birds dying from LPAI early in the outbreak, which would mean detection would occur primarily by collecting a sample from the infected bird, or two in the daily mortality. The instances where those initially dying birds pass while not shedding very much virus could account for the higher Ct values estimated at that point in the outbreak. Overall, the Ct simulation model requires further validation with field data where infected flocks are monitored prior to marketing.

The virus shedding model has several potential applications for informing risk management during an LPAI outbreak. For example, the Ct component could improve estimates for the time of virus introduction from diagnostic test results, especially when few results are available [12]. Nickbakhsh et al. (2013) included a transmission term in their within flock HPAI transmission model for spread due to susceptible bird contacts with infectious fecal contaminated dust or equipment [47]. The output from the virus shedding model in this analysis could be similarly used to model transmission due to environmental contamination in a LPAI transmission model. Furthermore, the virus shedding model could be used to adjust the transmission rate according to the amount of virus shed by a bird over time, as opposed to a constant rate across the bird’s infectious period, which is the current assumption in the model.

## Figures and Tables

**Table 1:** Input parameters for the Within House Turkey Tom LPAI Transmission Model.

Parameter Description	Distribution/Value	References
Adequate contact rate, the parameter that determines the rate of disease transmission	~Uniform (min = 0.5, max = 4.0)	[12]
Latent period length distribution	~Gamma (shape = 2.58, scale = 0.24); mean = 0.63 days, standard deviation = 0.39 days	[9,14,15]
Infectious period length distribution	~Gamma (shape = 4.04, scale = 2.92); mean = 11.78 days, standard deviation = 5.86 days	[8,9,13,14,15,16]
Time from infection to seroconversion distribution	~Gamma (shape = 3.56, scale = 1.63); mean = 5.80 days, standard deviation = 3.07 days	[17,18,19,20]
Proportion of infected birds that die	0.01	[12]
Proportion of infected birds that seroconvert	0.99	[8]

**Table 2:** Transmission Model Output by Seroprevalence. Median and 95% prediction interval for the number of days post virus exposure, infection prevalence in the house, and proportion of oral and cloacal shedding that already occurred, simulated from the Spackman et al. (2010), and Iqbal et al. (2012) data when certain levels of seroprevalence were first reached, as estimated from 6000 disease transmission model iterations [8,9].

Seroprevalence	Number of Days Post Virus Exposure	Prevalence of Infectious Birds	Proportion of Total Oral Shedding Already Occurred (Spackman et al. (2010) Data [8])	Proportion of Cloacal Shedding Already Occurred (Spackman et al. (2010) Data [8])	Proportion of Total Oral Shedding Already Occurred (Iqbal et al. (2012) Data [9])	Proportion of Cloacal Shedding Already Occurred (Iqbal et al. (2012) Data [9])
10%	11 (8–22)	0.88 (0.39–0.97)	0.46 (0.23–0.72)	0.02 (0.01–0.06)	0.30 (0.20–0.36)	0.07 (0.03–0.08)
20%	12 (9–24)	0.96 (0.59–0.98)	0.65 (0.40–0.86)	0.05 (0.02–0.13)	0.53 (0.38–0.60)	0.12 (0.10–0.17)
30%	12 (10–25)	0.95 (0.70–0.97)	0.77 (0.53–0.93)	0.09 (0.03–0.20)	0.71 (0.54–0.77)	0.21 (0.18–0.27)
40%	13 (10–27)	0.93 (0.75–0.94)	0.84 (0.63–0.96)	0.13 (0.05–0.28)	0.84 (0.66–0.88)	0.31 (0.28–0.37)
50%	14 (11–28)	0.90 (0.75–0.91)	0.89 (0.71–0.98)	0.20 (0.08–0.38)	0.91 (0.77–0.94)	0.43 (0.39–0.48)
60%	15 (12–29)	0.85 (0.73–0.87)	0.93 (0.78–0.99)	0.29 (0.13–0.49)	0.95 (0.85–0.96)	0.57 (0.53–0.61)
70%	16 (13–30)	0.79 (0.67–0.81)	0.96 (0.85–1.00)	0.41 (0.22–0.62)	0.98 (0.91–0.98)	0.71 (0.68–0.74)
80%	17 (14–32)	0.70 (0.58–0.73)	0.98 (0.91–1.00)	0.58 (0.37–0.77)	0.99 (0.95–0.99)	0.84 (0.81–0.86)
90%	19 (16–35)	0.55 (0.43–0.58)	1.00 (0.96–1.00)	0.81 (0.64–0.93)	1.00 (0.98–1.00)	0.96 (0.93–0.97)
98%	24 (21–42)	0.23 (0.13–0.27)	1.00 (1.00–1.00)	1.00 (0.98–1.00)	1.00 (1.00–1.00)	1.00 (1.00–1.00)

**Figure A1:**
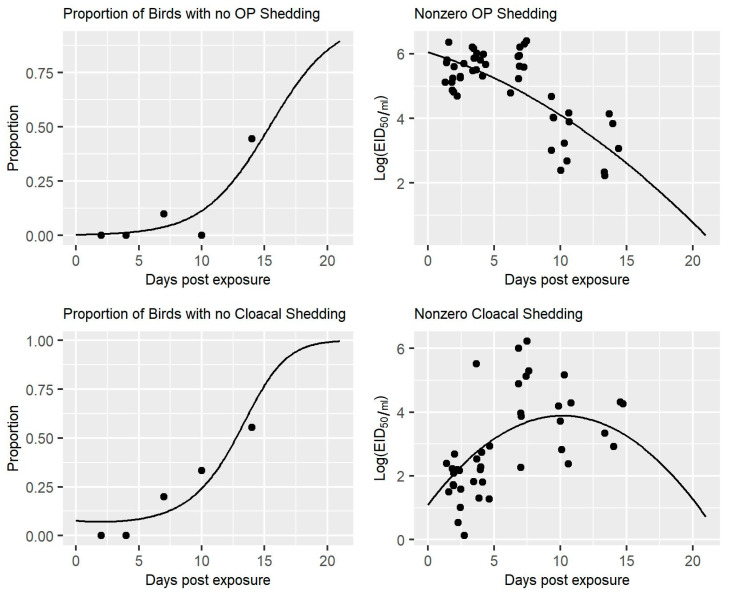
OP and Cloacal Swab Two-part Models for LPAIV H7N9 Strain. Estimated two-part models for OP and cloacal swabs from turkeys inoculated with the A/ruddy turnstone/DE/1538 2000 LPAIV H7N9 strain. Note that the nonzero shedding datapoints have been jittered for clarity.

## Data Availability

The data used in this study are available in Spackman et al. (2010) and Iqbal et al. (2012) [8,9].

## Appendix A

Two-part models were fit to the $log_{10}\;EID_{50}/ml$ values estimated from oropharyngeal (OP) and cloacal swabs sampled from turkeys inoculated with twelve different H7 low pathogenicity avian influenza virus (LPAIV) strains by Spackman et al. (2010) [8,22]. The OP and cloacal swabs were sampled from turkeys 2, 4, 7, 10, and 14 days post inoculation [8]. In two-part models, the data is treated as a mixture of the zero and nonzero components [22]. Here, the components were modeled using generalized linear mixed models with virus strain as the random effect [23]. An individual bird random effect was not evaluated due to data limitations.

Let $y_{ij}$ be the $log_{10}\;EID_{50}/ml$ observed for bird *i* inoculated with virus *j*; $\beta_{0}$, $\beta_{1}$, and $\beta_{2}$ the fixed effects; and $b_{0j}$ the random intercept, and $b_{1j}$ the random slope for virus *j* with $\left\{b_{0j},b_{1j}\right\}\;\sim \;N\left(0,D\right)$, where D is the variance-covariance matrix. Let $D=\left[\begin{array}{cc} d_{1} & d_{12}\\ d_{12} & d_{2} \end{array}\right]$ where $d_{1}$ is the standard deviation for $b_{0j}$, $d_{2}$ is the standard deviation for $b_{1j}$, and $d_{12}$ is the covariance. Three regression models were evaluated for the probability that virus was not detected from an OP swab sampled from bird *i* inoculated with virus strain *j* at time $t_{ij}$:

(A1) $$logit\left\{P\left(y_{i}=0\right)\right\}=\beta_{0}+\beta_{1}t_{i}$$

(A2) $$logit\left\{P\left(y_{ij}=0\right)\right\}=\beta_{0}+\beta_{1}t_{ij}+b_{0j}$$

(A3) $$logit\left\{P\left(y_{ij}=0\right)\right\}=\beta_{0}+\beta_{1}t_{ij}+b_{0j}+b_{1j}t_{ij}$$

Similarly, three regression models were evaluated for the probability that virus was not detected from a cloacal swab sampled from bird *i* inoculated with virus strain *j* at time $t_{ij}$:

(A4) $$logit\left\{P\left(y_{i}=0\right)\right\}=\beta_{0}+\beta_{1}t_{i}+\beta_{2}t_{i}^{2}$$

(A5) $$logit\left\{P\left(y_{ij}=0\right)\right\}=\beta_{0}+\beta_{1}t_{ij}+\beta_{2}t_{ij}^{2}+b_{0j}$$

(A6) $$logit\left\{P\left(y_{ij}=0\right)\right\}=\beta_{0}+\beta_{1}t_{ij}+\beta_{2}t_{ij}^{2}+b_{0j}+b_{1j}t_{ij}$$

Next, let $\beta_{0}^{*}$, $\beta_{1}^{*}$, and $\beta_{2}^{*}$ be the fixed effects; $b_{0j}^{*}$ the random intercept, and $b_{1j}^{*}$ the random slope for virus *j* with $\left\{b_{0j}^{*},b_{1j}^{*}\right\}\;\sim \;N\left(0,\;D^{*}\right)$, where $D^{*}$ is the variance-covariance matrix; and $\varepsilon_{ij}$ the random error with $\varepsilon_{ij}\;\sim \;N\left(0,\;\sigma^{2}\right)$. Let $D^{*}=\left[\begin{array}{cc} d_{1}^{*} & d_{12}^{*}\\ d_{12}^{*} & d_{2}^{*} \end{array}\right]$ where $d_{1}^{*}$ is the standard deviation for $b_{0j}^{*}$, $d_{2}^{*}$ is the standard deviation for $b_{1j}^{*}$, and $d_{12}^{*}$ is the covariance. The same three regression model formulations were evaluated for OP and cloacal swabs for the virus amount estimated from the swab at time $t_{ij}$, given this amount was not zero:

(A7) $$y_{i}|y_{i}>0=\beta_{0}^{*}+\beta_{1}^{*}t_{i}+\beta_{2}^{*}t_{i}^{2}+\varepsilon_{i}$$

(A8) $$y_{ij}|y_{ij}>0=\beta_{0}^{*}+\beta_{1}^{*}t_{ij}+\beta_{2}^{*}t_{ij}^{2}+b_{0j}^{*}+\varepsilon_{ij}$$

(A9) $$y_{ij}|y_{ij}>0=\beta_{0}^{*}+\beta_{1}^{*}t_{ij}+\beta_{2}^{*}t_{ij}^{2}+b_{0j}^{*}+b_{1j}^{*}t_{ij}+\varepsilon_{ij}$$

The optimal regression equations were selected using Akaike’s Information Criterion (AIC) [27]. The AIC values are given in Table A1. The regression equation with the smallest AIC (indicating best fit) was selected for each swab type and two-part model component. Burnham and Anderson (2002) outline a rule of thumb for comparing AIC values, where differences greater than 10 provide substantial support in favor of the model with the smaller AIC, differences between 4 and 7 provide less support, and differences between 0 and 2 do not provide strong evidence in favor of either model [28].

Equation (A2) was selected to estimate the probability that virus was not detected from an OP swab sample. Though Equation (A2) had the lowest AIC value, the difference was less than 10 compared to the AIC values estimated from the other two regression models. Thus, we do not have conclusive evidence that Equation (A2) is the best fitting model. Under Equation (A2), the log odds that virus was not detected from an OP swab initially following infection is dependent on the virus strain. The subsequent increase in the log odds of not detecting virus is the same across strains, due to the absence of the random slope term. Therefore, by using Equation (A2), we assume in the simulation model that birds stop shedding orally at the same rate across the different virus strains. The $\beta_{0}$ parameter represents the general log odds that a bird does not shed virus immediately following infection. The log odds that a bird does not shed virus at time 0 are modified according to the virus strain as captured by the random intercept term $b_{0j}$. However, the two-part models were not used to simulate virus shedding in the disease transmission model until birds transitioned out of the latent period, which was 0.63 days long on average. The increase over time in the log odds that a bird does not shed virus increases according to the slope term $\beta_{1}$.

**Table A1:** Two-part Regression Model Comparisons. AIC values of the regression models estimated from the zero and nonzero portions of the OP and cloacal swab data from Spackman et al. (2010) [8].

Swab Type	Two-Part Model Component	Regression Equation	AIC
Oropharyngeal	$P\left(y_{ij}=0\right)$	Equation (A1)	251.28
		Equation (A2)	243.50
		Equation (A3)	247.10
	$y_{ij}|y_{ij}>0$	Equation (A7)	1391.75
		Equation (A8)	1225.04
		Equation (A9)	1183.44
Cloacal	$P\left(y_{ij}=0\right)$	Equation (A4)	806.08
		Equation (A5)	758.90
		Equation (A6)	742.60
	$y_{ij}|y_{ij}>0$	Equation (A7)	980.74
		Equation (A8)	938.63
		Equation (A9)	934.86

Equation (A9) was selected to estimate the amount of virus detected by the OP swab sample, given this amount was not zero. The pattern observed for the nonzero OP swab portion was high amounts of virus initially that decreased over time. There was substantial support in favor of Equation (A9), as there was a difference of greater than 10 compared to the next smallest AIC value. This provides evidence that the amount of virus initially shed orally by birds, and the subsequent decrease in the amount of virus shed over time varies by virus strain. The $\beta_{0}^{*}$ parameter represents a general intercept term for the amount of virus shed by birds at time 0. This amount is modified by the strain-specific random effect $b_{0j}^{*}$. The $\beta_{1}^{*}$ and $\beta_{2}^{*}$ parameters determine the decrease in the amount of virus shed over time. The significance of the quadratic term suggests that the rate of decrease is faster as time increases. The linear decrease in the amount of virus over time increases or decreases for a virus strain according to the random slope term $b_{1j}^{*}$. Lastly, $\varepsilon_{ij}$ models the variation in the amount of virus shed by individual birds infected with a given virus strain.

Equation (A6) was selected to estimate the probability that virus was not detected from a cloacal swab. There was considerable variation between virus strains observed in the data for the initial probability that virus was not detected, as well as the subsequent probabilities over time: for two strains, there was a lower probability initially that then increased, whereas for the other strains, the probability decreased from a higher initial value before increasing. These patterns occurred at different rates across virus strains. Consequently, the best fit model included both a random intercept and slope term for the virus strain.

Equation (A9) was selected to estimate the amount of virus detected by a cloacal swab, given the amount was not zero. Random intercept and slope terms for the virus strain were included in this model. The pattern observed in the data was parabolic: the amount of virus increased post infection, peaked after several days, and then decreased. The difference in the AIC values for Equation (A9) and the next best fitting model, Equation (A8), which did not include a random intercept term for virus, was 3.77. Therefore, we do not have overwhelming evidence that the rate of change in the amount of virus detected by cloacal swabs over time varied by virus strain. The interpretation of the parameters for the cloacal swab two-part model is similar to the interpretations discussed for the OP swab two-part model.

Overall, the two-part model results for the OP and cloacal swabs suggest that there is variation between LPAIV strains in the proportion of birds that shed virus, and the amount of virus shed by the birds over time post infection. However, the nature of this variation was not always clear. There were differences of less than 10 between the AIC values of best fitting models for the probability that virus was not detected by OP swabs, and the amount of virus detected from cloacal swabs, given this amount was not zero. More information on the effect of strain on virus shedding could be gleaned by fitting two-part models to data from other LPAIV inoculation experiments.

The parameters for Equations (A2) and (A6) were estimated by maximum likelihood (Laplace approximation) using the ‘glmer’ function from the lme4 package in R statistical software [29,40]. The mean estimates and 95% confidence intervals (CI) are given in Table A2 for Equation (A2), estimated from the OP swab data; and Table A3 for Equation (A6), estimated from the cloacal swab data. The 95% CI were estimated using the bootstrap method from the ‘confint’ function in R [29]. The parameters for Equation (A9) were estimated from the OP and cloacal swab data by maximum likelihood using the ‘lme’ function from the nlme package in R [30]. The mean estimates and 95% CI are given in Table A4 for Equation (A9), estimated from the OP swab data; and in Table A5 for Equation (A9), estimated from the cloacal swab data. The 95% CI were estimated using the ‘intervals’ function in R, which uses a normal approximation to the distribution of the maximum likelihood estimators [30].

**Table A2:** Regression Model Estimates for OP Swab Zero Component. Parameter estimates for the probability of observing no viral shedding over time from a turkey sampled with an OP swab modeled by regression Equation (A2).

Parameter	Description	Mean (95% CI)
$\beta_{0}$	Fixed intercept	−5.88 (−7.57, −4.77)
$\beta_{1}$	Fixed coefficient for the number of days post exposure	0.38 (0.29, 0.50)
$d_{1}$	Random intercept standard deviation	0.96 (0.15, 1.55)

**Table A3:** Regression Model Estimates for Cloacal Swab Zero Component. Parameter estimates for the probability of observing no viral shedding over time from a turkey sampled with a cloacal swab modeled by regression Equation (A6).

Parameter	Description	Mean (95% CI)
$\beta_{0}$	Fixed intercept	−0.42 (−1.74, 0.82)
$\beta_{1}$	Fixed coefficient for the number of days post exposure	−0.22 (−0.47, 0.02)
$\beta_{2}$	Fixed coefficient for the number of days post exposure squared	0.02 (0.01, 0.04)
$d_{1}$	Random intercept standard deviation	1.78 (0.81, 2.68)
$d_{2}$	Random slope standard deviation	0.17 (0.07, 0.27)
$\rho_{12}$	Random intercept and random slope correlation	−0.83 (−0.99, −0.28)

**Table A4:** Regression Model Estimates for OP Swab Nonzero Component. Parameter estimates for the amount of virus observed from a turkey sampled with an OP swab over time, given the amount is not zero. This amount was modeled by regression Equation (A9).

Parameter	Description	Mean (95% CI)
$\beta_{0}^{*}$	Fixed intercept	5.13 (4.47, 5.79)
$\beta_{1}^{*}$	Fixed coefficient for the number of days post exposure	−0.18 (−0.28, −0.08)
$\beta_{2}^{*}$	Fixed coefficient for the number of days post exposure squared	−0.007 (−0.013, −0.001)
σ	Standard deviation of the normal distribution for the errors	0.88 (0.82, 0.94)
$d_{1}^{*}$	Random intercept standard deviation	1.07 (0.71, 1.59)
$d_{2}^{*}$	Random slope standard deviation	0.10 (0.07, 0.16)
$\rho_{12}^{*}$	Random intercept and random slope correlation	−0.80 (−0.94, −0.45)

**Table A5:** Regression Model Estimates for Cloacal Swab Nonzero Component. Parameter estimates for the amount of virus observed from a turkey sampled with a cloacal swab over time, given the amount is not zero. This amount was modeled by regression Equation (A9).

Parameter	Description	Mean (95% CI)
$\beta_{0}^{*}$	Fixed intercept	0.84 (0.34, 1.33)
$\beta_{1}^{*}$	Fixed coefficient for the number of days post exposure	0.47 (0.31, 0.62)
$\beta_{2}^{*}$	Fixed coefficient for the number of days post exposure squared	−0.03 (−0.04, −0.02)
σ	Standard deviation of the normal distribution for the errors	1.21 (1.12, 1.31)
$d_{1}^{*}$	Random intercept standard deviation	0.30 (0.10, 0.93)
$d_{2}^{*}$	Random slope standard deviation	0.06 (0.03, 0.13)
$\rho_{12}^{*}$	Random intercept and random slope correlation	0.64 (−0.95, 1.00)

The strain-specific estimates for the two-part models are given in Table A6. One of the strain-specific two-part models was randomly selected for each iteration to simulate oral and cloacal shedding in the LPAIV transmission model for the Spackman et al., 2010 scenario [8]. As a result, variance due to virus strain when birds stop shedding, and the amounts of virus they shed are captured in this scenario.

**Table A6:** Estimated Strain-specific Two-part Models for OP and Cloacal Swabs.

LPAIV Strain	Strain-Specific Two-Part Model for OP and Cloacal (CL) Swabs
A/chicken/MD/MinhMa/2004 (H7N2)	OP: $logit\left\{P\left(y_{ij}=0\right)\right\}=-5.14+0.38t_{ij}\;E\left[y_{ij}|y_{ij}>0\right]=5.36-0.30t_{ij}-0.007t_{ij}^{2}$ CL: $logit\left\{P\left(y_{ij}=0\right)\right\}=0.13-0.09t_{ij}+0.02t_{ij}^{2}\;E\left[y_{ij}|y_{ij}>0\right]=0.67+0.43t_{ij}-0.03t_{ij}^{2}$
A/chicken/NJ/15086-3/1994 (H7N3)	OP: $logit\left\{P\left(y_{ij}=0\right)\right\}=-7.00+0.38t_{ij}\;E\left[y_{ij}|y_{ij}>0\right]=3.91-0.07t_{ij}-0.007t_{ij}^{2}$ CL: $logit\left\{P\left(y_{ij}=0\right)\right\}=-0.24-0.46t_{ij}+0.02t_{ij}^{2}\;E\left[y_{ij}|y_{ij}>0\right]=0.90+0.48t_{ij}-0.03t_{ij}^{2}$
A/chicken/NY/12273-11/1999 (H7N3)	OP: $logit\left\{P\left(y_{ij}=0\right)\right\}=-6.61+0.38t_{ij}\;E\left[y_{ij}|y_{ij}>0\right]=4.48-0.15t_{ij}-0.007t_{ij}^{2}$ CL: $logit\left\{P\left(y_{ij}=0\right)\right\}=0.03-0.19t_{ij}+0.02t_{ij}^{2}\;E\left[y_{ij}|y_{ij}>0\right]=0.50+0.40t_{ij}-0.03t_{ij}^{2}$
A/chicken/NY/30749-3/2000 (H7N2)	OP: $logit\left\{P\left(y_{ij}=0\right)\right\}=-5.66+0.38t_{ij}\;E\left[y_{ij}|y_{ij}>0\right]=7.01-0.28t_{ij}-0.007t_{ij}^{2}$ CL: $logit\left\{P\left(y_{ij}=0\right)\right\}=0.21-0.01t_{ij}+0.02t_{ij}^{2}\;E\left[y_{ij}|y_{ij}>0\right]=1.24+0.54t_{ij}-0.03t_{ij}^{2}$
A/chicken/NY/3112-1/1995 (H7N2)	OP: $logit\left\{P\left(y_{ij}=0\right)\right\}=-5.66+0.38t_{ij}\;E\left[y_{ij}|y_{ij}>0\right]=3.58-0.02t_{ij}-0.007t_{ij}^{2}$ CL: $logit\left\{P\left(y_{ij}=0\right)\right\}=-0.09-0.31t_{ij}+0.02t_{ij}^{2}\;E\left[y_{ij}|y_{ij}>0\right]=0.74+0.45t_{ij}-0.03t_{ij}^{2}$
A/chicken/PA/9801289/1998 (H7N2)	OP: $logit\left\{P\left(y_{ij}=0\right)\right\}=-4.59+0.38t_{ij}\;E\left[y_{ij}|y_{ij}>0\right]=5.74-0.28t_{ij}-0.007t_{ij}^{2}$ CL: $logit\left\{P\left(y_{ij}=0\right)\right\}=-0.15-0.37t_{ij}+0.02t_{ij}^{2}\;E\left[y_{ij}|y_{ij}>0\right]=0.78+0.44t_{ij}-0.03t_{ij}^{2}$
A/guinea hen/MA/148081-11/2002 (H7N2)	OP: $logit\left\{P\left(y_{ij}=0\right)\right\}=-6.39+0.38t_{ij}\;E\left[y_{ij}|y_{ij}>0\right]=6.42-0.30t_{ij}-0.007t_{ij}^{2}$ CL: $logit\left\{P\left(y_{ij}=0\right)\right\}=0.20-0.02t_{ij}+0.02t_{ij}^{2}\;E\left[y_{ij}|y_{ij}>0\right]=0.74+0.42t_{ij}-0.03t_{ij}^{2}$
A/mallard/OH/421/1987 (H7N8)	OP: $logit\left\{P\left(y_{ij}=0\right)\right\}=-6.33+0.38t_{ij}\;E\left[y_{ij}|y_{ij}>0\right]=3.99-0.12t_{ij}-0.007t_{ij}^{2}$ CL: $logit\left\{P\left(y_{ij}=0\right)\right\}=-0.14-0.36t_{ij}+0.02t_{ij}^{2}\;E\left[y_{ij}|y_{ij}>0\right]=0.59+0.42t_{ij}-0.03t_{ij}^{2}$
A/pintail/MN/423/1999 (H7N3)	OP: $logit\left\{P\left(y_{ij}=0\right)\right\}=-5.10+0.38t_{ij}\;E\left[y_{ij}|y_{ij}>0\right]=4.98-0.15t_{ij}-0.007t_{ij}^{2}$ CL: $logit\left\{P\left(y_{ij}=0\right)\right\}=-0.06-0.28t_{ij}+0.02t_{ij}^{2}\;E\left[y_{ij}|y_{ij}>0\right]=1.22+0.53t_{ij}-0.03t_{ij}^{2}$
A/ruddy turnstone/DE/1538/2000 (H7N9)	OP: $logit\left\{P\left(y_{ij}=0\right)\right\}=-5.86+0.38t_{ij}\;E\left[y_{ij}|y_{ij}>0\right]=6.06-0.13t_{ij}-0.007t_{ij}^{2}$ CL: $logit\left\{P\left(y_{ij}=0\right)\right\}=0.13-0.09t_{ij}+0.02t_{ij}^{2}\;E\left[y_{ij}|y_{ij}>0\right]=1.09+0.55t_{ij}-0.03t_{ij}^{2}$
A/turkey/NY/4450-4/1994 (H7N2)	OP: $logit\left\{P\left(y_{ij}=0\right)\right\}=-6.88+0.38t_{ij}\;E\left[y_{ij}|y_{ij}>0\right]=4.42-0.09t_{ij}-0.007t_{ij}^{2}$ CL: $logit\left\{P\left(y_{ij}=0\right)\right\}=-0.17-0.39t_{ij}+0.02t_{ij}^{2}\;E\left[y_{ij}|y_{ij}>0\right]=1.02+0.49t_{ij}-0.03t_{ij}^{2}$
A/turkey/VA/SEP-67/2002 (H7N2)	OP: $logit\left\{P\left(y_{ij}=0\right)\right\}=-4.78+0.38t_{ij}\;E\left[y_{ij}|y_{ij}>0\right]=5.64-0.29t_{ij}-0.007t_{ij}^{2}$ CL: $logit\left\{P\left(y_{ij}=0\right)\right\}=0.07-0.15t_{ij}+0.02t_{ij}^{2}\;E\left[y_{ij}|y_{ij}>0\right]=0.54+0.42t_{ij}-0.03t_{ij}^{2}$

The regression models estimated from the nonzero values were assessed by plotting the Pearson residuals versus the fitted values for the overall fit, as well as the fit for each virus strain. The assumption of normal distributed within-group errors was assessed with a normal quantile-quantile (Q-Q) plot of the residuals for each virus strain. Similarly, the assumption of normal distributed random effects was assessed with a normal Q-Q plot of the predicted random effects for the regression models estimated from both the zero and nonzero portions of the data [23,24,25,26]. The regression fits could also be viewed directly since, as defined, the response variable varied only by time. An example is given in Figure A1 for the H7N9 LPAIV strain, A/ruddy turnstone/DE/1538/2000. The residuals of the regression models estimated from the nonzero portion of the data were fairly large (>2) at 14 days post-inoculation for some strains. The evidence of issues at 14 days post-inoculation was unsurprising, as few birds were shedding at that point in the experiment [8]. As such, the predicted amounts of shedding greater than two weeks post-inoculation should be viewed with caution.
